# Effects of experimental warming on two tropical Andean aquatic insects

**DOI:** 10.1371/journal.pone.0271256

**Published:** 2022-07-27

**Authors:** Silvana Gallegos-Sánchez, Eduardo Domínguez, Andrea C. Encalada, Blanca Ríos-Touma

**Affiliations:** 1 Instituto de Biodiversidad Neotropical (IBN), Consejo Nacional de Investigaciones Científicas y Técnicas (CONICET), Facultad de Ciencias Naturales e Instituto Miguel Lillo, Universidad Nacional de Tucumán, Yerba Buena, Tucumán, Argentina; 2 Grupo de Investigación en Biodiversidad, Medio Ambiente y Salud (BIOMAS), Universidad de Las Américas, Quito, Ecuador; 3 Colegio de Ciencias Biológicas y Ambientales COCIBA, Instituto BIOSFERA-USFQ, Universidad San Francisco de Quito USFQ, Quito, Ecuador; Universitat de Barcelona, SPAIN

## Abstract

Temperatures have increased around the globe, affecting many ecosystems, including high-elevation Andean streams where important aquatic insect species coexist. Depending on the magnitude of change, warming could lead to the mortality of sensitive species, and those tolerant to rising water temperatures may exhibit differences in growth rates and development. Taxon-specific optimal temperature ranges for growth determine how high or low temperatures alter an organism’s body size. In this study, we observed the effects of different climate change scenarios (following three scenarios of the 2021 IPCC predictions) in two aquatic insect species distributed in high-elevation streams in Ecuador: the mayfly *Andesiops peruvianus* (Ephemeroptera: Baetidae) and the caddisfly *Anomalocosmoecus illiesi* (Trichoptera: Limnephilidae). We assessed how increased water temperatures affect larval growth rates and mortality during a 10-day microcosm experiment. Our results showed that *Andesiops peruvianus* was more thermally sensitive than *Anomalocosmoecus illiesi*. Mortality was higher (more than 50% of the individuals) in mayflies than in caddisflies, which presented mortality below 12% at +2.5°C and +5°C. Mortality in mayflies was related to lower dissolved oxygen levels in increased temperature chambers. Higher temperatures affected body size and dry mass with a faster growth rate of *Andesiops peruvianus* larvae at experimentally higher temperatures, suggesting an important response of this hemimetabolous species to stream temperatures. For *Anomalocosmoecus illiesi*, we did not find significant changes in mortality, body size or growth rate in response to temperature changes during our experiment. *In situ* outcomes of species survival and growth in Andean streams are difficult to predict. Nevertheless, our results suggest that at only +2.5°C, a water temperature increase affected the two insect taxa differentially, leading to a drastic outcome for one species’ larvae while selecting for a more tolerant species. Our study suggests that climate change might produce significant mortality and growth rate effects on ectotherm tropical aquatic insects, especially Andean mayflies, which showed higher sensitivity to increased water temperature scenarios.

## Introduction

Temperatures have increased globally due to the rise of greenhouse gases from humanmade activities since the last century [1]. All types of ecosystems have been affected, including continental freshwater ecosystems. It is clear that in these new temperature scenarios, species physiology and behaviors, as well as the composition, diversity, and functioning of freshwater habitats, are also changing [2]. Regarding the species level response, three universal responses of organisms to warming temperatures have been suggested: a) shifting their distributions (in terms of latitude or elevation); b) changes in species phenology (in time/season); and c) changes (reduction) in body size [3–7]. The responses by species will define how they cope with new environmental conditions that will arise with climate change events [8].

Species body size reflects natural selection, promoting their adaptation to specific local environmental conditions and further influencing their life histories, home range, and fitness [9]. Reductions in body size in response to global warming have attracted considerable attention. In ectotherms, body size is primarily controlled by temperature, which is a central ecological factor in most physiological processes [10–12]. Temperature dictates an ectotherm’s metabolic rate and can influence ontogeny, development, and growth [13] as well as other processes, such as respiration, fecundity, and phenology [14]. These temperature-influenced aspects are ecologically linked to body size, as they control how populations grow and persist in ecosystems. Therefore, significant alterations in a population’s body size due to increasing temperatures could reflect changes to downstream population-wide aspects such as their abundance, overall community composition, and ecological interactions, such as competition and predation [15].

Adult body size is the result of growth rate (increase in body size) and development rate (changes in life stages) [12, 16]. In some ectotherms (e.g., crustaceans), rearing temperature influences growth rates more than development rates, especially at early larval stages [17]. However, this is not always the case, as other studies have found a much greater influence of warmer temperatures on development rates than on growth rates [16]. For example, other taxonomic groups of ectotherms, such as insects (terrestrial and aquatic), fish, and lizards, have shown that organisms grow larger in cool versus warm environments [18–21]. From these observations, size may be tightly correlated with an optimal temperature at which organisms grow, defined by Atkinson (1994) as “the temperature size rule” (TSR). Most experiments conducted to date have employed controlled environments where organisms demonstrate temperature-influenced differences in their metabolic rate, resulting in differences in growth [19].

Studies that have analyzed the life histories of aquatic insects from temperate regions show that some follow the TSR; for instance, growth rates are typically slower and mature individuals are larger in cold environments [22–24]. In addition to temperature, for the mayfly *Hexagenia* spp., food quantity and quality interact with temperature to constrain body size [25]. In contrast, experiments performed with two holometabolous aquatic insects (i.e., a trichopteran *Hydropsyche betteni* and a black fly *Simulium vittatum*) found slower or stagnant growth rates at colder temperatures regardless of food quality/quantity. However, growth rates were faster at higher temperatures in both species but dependent on the food type [26]. A field comparison study between a caddisfly (*Hydropsyche oslari*) and two mayflies (*Drunella flavilinea* and *Serratella tibialis*) showed that river temperature fluctuations affected species growth in different ways, suggesting that growth was more related to changes in food availability than temperature [27]. In addition, recent studies addressing other factors, such as oxygen consumption, found that oxygen demand set thermal limits and modified body size in *Dinocras cephalotes*, a stonefly from Europe [28]. Additionally, aspects such as juvenile mortality of insects were highly correlated with rising temperatures and limited oxygen [7, 29].

Higher temperatures and changes in climate scenarios have been predicted to significantly affect tropical high Andes rivers and streams [1, 30, 31]. These ecosystems are important habitats for many diverse, unique, and endemic stream fauna and flora, which provide critical ecosystem services [32, 33]. Some of the most dominant fauna in these high-elevation streams are larvae of aquatic insects that spend most of their life cycle in these specific habitats [34]. Aquatic insect larvae are frequently used as bioindicators of water quality in Andean streams due to their sensitivity to environmental changes (e.g., land-use change, pollution) [35–37]. Despite their ecological significance in tropical high-elevation rivers, there are still many gaps in our knowledge of the taxonomy of aquatic insects, life histories, behavior, and ecology [35, 38, 39]. Specifically, few studies have addressed how these groups respond to global warming (e.g., range shift, phenological shifts, and metabolic plasticity) [34]. In general, aquatic insects in the tropical Andes have narrow thermal tolerances compared with their temperate counterparts and show lower dispersal capabilities, making them more vulnerable to climate change [40–43].

The environmental conditions in the tropics greatly influence the life histories of aquatic insects [39]. Both temperature and precipitation are important factors that can influence growth rates and development [39, 44]. Tropical lowland areas with warmer temperatures have more multivoltine aquatic insect species, and size classes might be dependent on seasonality [45–49]. Similarly, the life histories of taxa distributed in high-elevation watercourses in the tropics showed plastic voltinism, synchronized with seasonality and subsequent availability of resources [46, 50, 51]. For example, a study of *Helycopsyche* spp. (a holometabolous caddisfly species) distributed in high-elevation streams in Ecuador found larger larvae in páramo areas than their counterparts from lower elevations that live in streams with an average 1°C higher temperature [50]. Hemimetabolous species, such as mayflies *Andesiops peruvianus* and *Meridialaris tintinnabula*, exhibited heterogeneous size classes in high-elevation mountains in Bolivia during a one-year study [46]. Sizes and growth in both hemimetabolous and holometabolous aquatic insects depended on temperature and hydrological conditions during larval development [46, 50, 52]. In terms of metabolism, it was observed that Baetidae mayflies had been seen to reduce their metabolic performance, show increased stress, and increase juvenile mortality when subjected to higher temperatures [53]. These effects are not universal in aquatic insects, for example, stoneflies [54]. Another study showed that mayflies *Andesiops* spp. distributed in high-elevation zones had higher acclimation ability than those from lower elevations, suggesting an enhanced ability to adapt to daily temperature swings common in high-elevation areas [53]. In summary, changes in water temperature can modify species growth rates and influence their biomass and, therefore, voltinism and reproduction [55–57]. However, the specific effects of increased temperature following predicted IPCC scenarios on development, growth rates, and body size in aquatic insects from the tropical Andes have not been addressed.

In this way, our study aimed to quantify the effects of global warming scenarios for two aquatic insect species: hemimetabolous *Andesiops peruvianus* (Ephemeroptera: Baetidae) and holometabolous *Anomalocosmoecus illiesi* (Trichoptera: Limnephilidae), species that are widely distributed in high-Andean watercourses. Based on previous findings where temperature affected growth rates and body size, we hypothesized that warmer scenarios would affect body sizes [12, 58, 59]. We specifically predicted that higher temperatures should affect both species, resulting in smaller body sizes, reduced dry mass, and accelerated growth rates than in colder conditions. Additionally, we predicted that increasing temperatures would affect juvenile stages (larval stage) by specifically altering development and survival. For the latter, we included these hemimetabolous and holometabolous insects as part of our experimental design to assess the effects of temperature-related developmental differences between different life histories.

## Materials and methods

### Experimental microcosm setup

**Fig 1:**
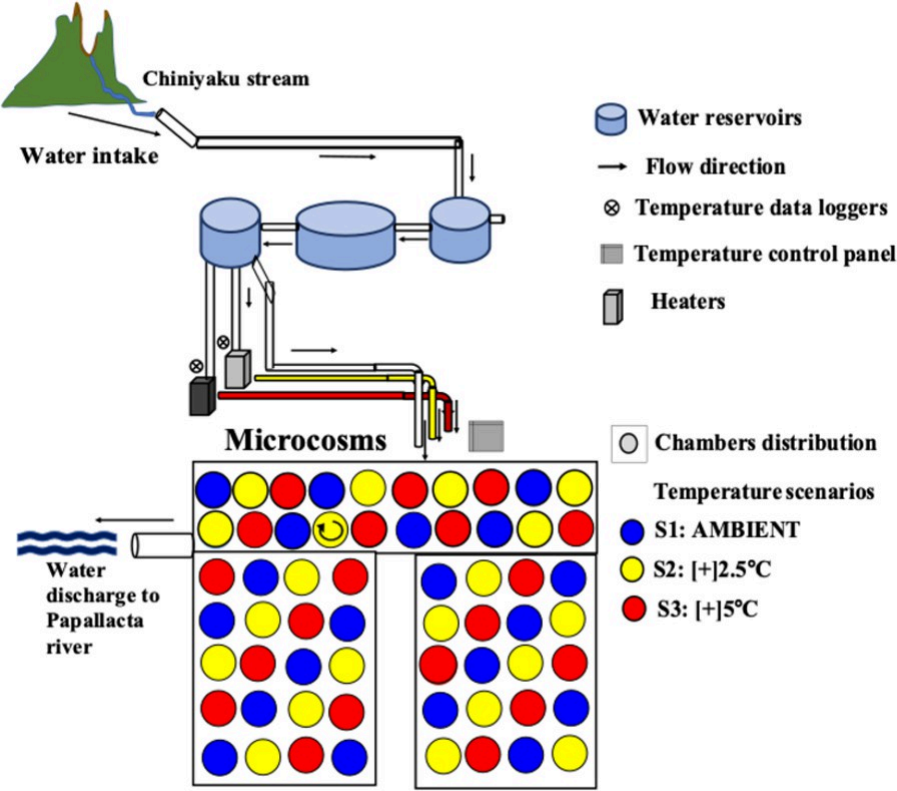
Microcosm. Diagram of the experimental facility from Laboratorio de Ecología Acuática (LEA), Universidad San Francisco de Quito (USFQ), located in Papallacta, Ecuador.

A 10-day microcosm experiment was performed from February 1^st^ to February 11^th^, 2019, in Papallacta, Napo, Ecuador. The microcosm facility consisted of sixty 2 L fiberglass chambers housed within a translucent greenhouse adjacent to the stream [60]. Chambers were constantly fed with water from the Chiniyaku stream (3380 m.a.s.l) (Fig 1), a tributary from the Papallacta River in the Napo subbasin and the Amazon Basin (Fig 3). Our study area’s average stream water temperature fluctuated from a low of 6°C to a high of 15°C. The experimental setup followed the natural variation in ambient stream temperatures, which were subsequently additionally heated following each of three scenarios (S1 = ambient temperature, S2 = increase of 2.5°C and S3 = increase of 5°C) of global warming [61, 62]. Daily stream ambient temperature information was sent from data loggers installed before stream water entered the system; heaters then warmed the ambient water temperature. Twenty chambers were heated at +2.5°C [scenario 2:*S2*] and twenty at +5°C [scenario 3: *S3*], while the remaining twenty were left as a control [scenario 1:*S1*] temperature (Fig 1)

**Fig 2:**
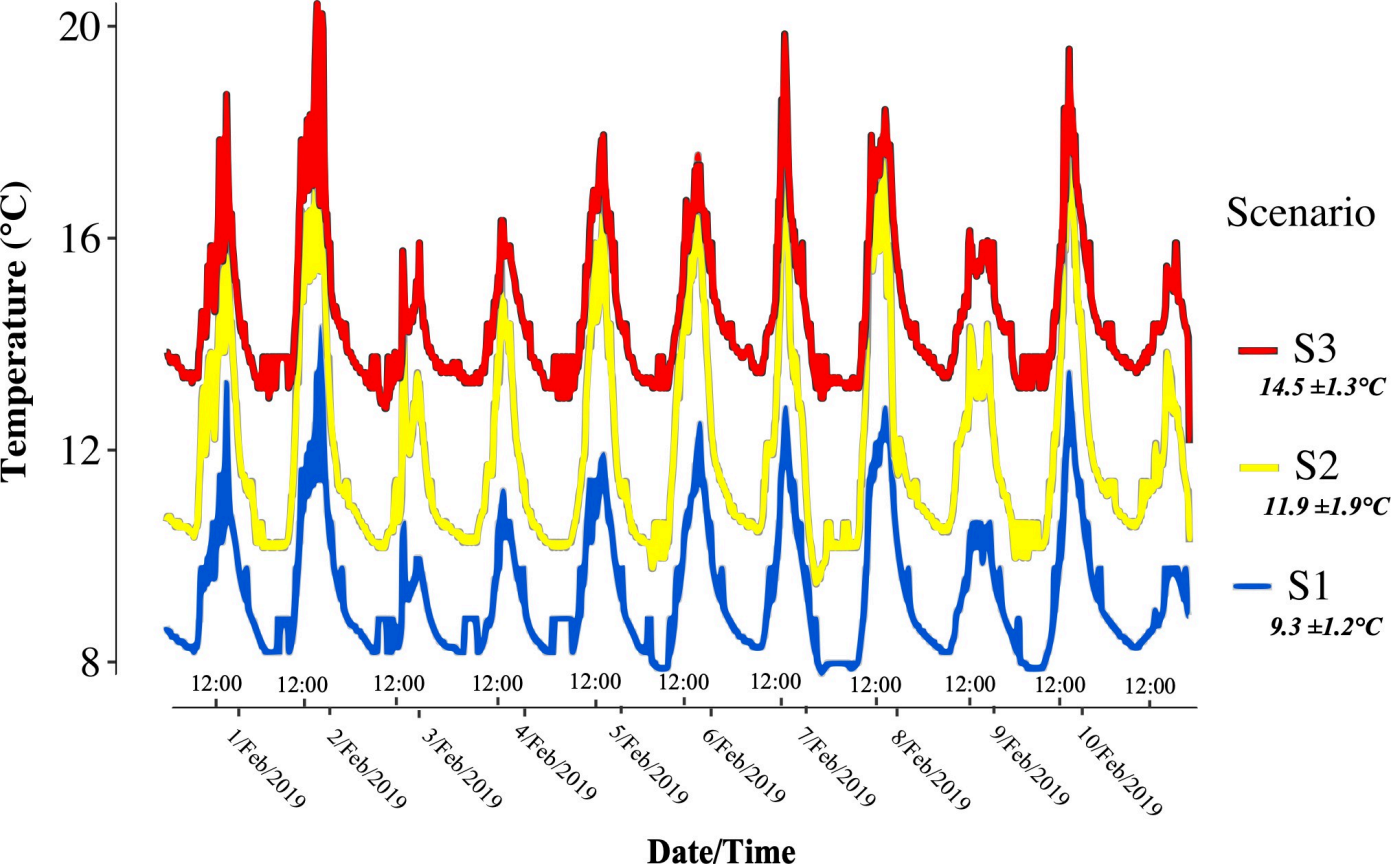
Water temperature during the microcosm experiment. HOBO data loggers recorded temperatures (1162 sampling points) from chambers in the control/ambient [*S1*] scenario (blue), +2.5°C [*S2*] scenario (yellow), and +5°C [*S3*] scenario (red) throughout the 10-day experiment. The X-axis shows recorded temperatures from the HOBO data logger with date and hour (12:00 pm). The Y-axis represents the temperatures in degrees Celsius (°C).

The microcosm facility was equipped with two heaters with an electronic control processor that raised the water temperature and distributed it into two separate pipes (Fig 1). Water pipes inside the microcosm facility were arranged circularly to maintain constant temperatures during the experiment. The temperature in each chamber was checked constantly throughout the experiment using portal thermometers. Before starting the experiment, we placed two data loggers (HOBO UA 8K, Onset, Bourne, USA) inside six chambers (two per temperature scenario): one closer to the heater and one at the furthest chamber to record water temperature. Data loggers were programmed to record water temperature every 15 minutes throughout the experiment. These recordings helped verify that the increasing temperature pattern was held consistently throughout the experiment. The average temperatures recorded from the HOBO data loggers were 9.3°C (SD = ±1.2) for S1, 11.9°C (SD = ±1.9) for S2, and 14.5 (SD = ±1.3) for S3 (Fig 2).

The ambient water temperature of the microcosm facility differed by ~1.2°C from the insect collection site [41]. Nevertheless, since our target species were distributed in both stream locations, we considered this temperature to be the natural variation range for their life cycle.

We chose to replicate three temperatures within the range predicted by the RCP8.5 emission scenario, where temperatures are expected to increase between 1.5°C and 5°C by the end of this century [1, 61]. We based this temperature design on projections from the fifth report of the IPCC and an analysis of the warming trend for the Andes, specifically in the mountains situated in the tropical zone [30, 61, 63]. Evidence suggests that high elevation is a factor that could enhance warming effects on mountain ecosystems [64]. In addition, projections suggest that unexpected extreme climatic conditions have already occurred in recent years and predict an increase in stronger heatwaves throughout all Andean ecosystems in the tropics [1, 65].

Three precolonized tiles with periphyton (35x35 mm) were set inside the chambers eight hours before starting the experiment. Tiles were previously placed in a channel with water from a nearby fishless Andean stream for approximately ten days to colonize with periphytic algae. All tiles had the same photoperiodic pattern during the days of colonization and served as a food source for the aquatic insects.

### Environmental tracking of the experiment

To assess water quality during the experiment, we collected water samples (7 AM Day 1) to analyze nitrates, phosphates, and ammonium in two random chambers for each temperature scenario. The samples were fixed with 5% sulfuric acid and stored at 4°C until they were analyzed. In addition, temperature, conductivity, pH, and total dissolved solids were measured with a HANNA HI 98130 multiparameter (St. Curepipe, Mauritius). Dissolved oxygen and (%) saturation were measured for the three temperature scenarios using a Milwaukee Mi605 oxygen meter (Rocky Mount, United States). These procedures were repeated on the fifth and tenth days of the experiment. Water samples were processed at laboratories of the Universidad de las Americas in Quito (UDLA), Ecuador. Nitrates, phosphates, and ammonia were analyzed following standard methods [66].

### Organic matter in the experimental microcosms

Andean rivers receive energy inputs from different sources. Organic matter is one of the main food resources for aquatic taxa and comprises coarse (CPOM) and fine particulate organic matter (FPOM) [67]. Previous studies showed that Neotropical macroinvertebrates from Andean streams tended to be generalists or at least combined two types of food sources, in which much of their content was FPOM [67, 68]. As the microcosm received water from a natural source, the Chiniyaku stream, it was important to measure the variance of fine particulate organic matter processed and transported to each chamber during the experiment, specifically if our target aquatic species depended on this type of resource. Samples of FPOM were collected on the first, fifth, and tenth day of the experiment from chambers in the microcosm. We followed the methods of Hutchens et al. (2017) where samples were filtered with 25 mm microfiber filters with a 1-micron pore size (Pall Corporation, Mexico). Each sample was placed inside an aluminum foil bag and frozen for storage. Filtration was performed at two random chambers from each temperature scenario, collecting samples from the water column. These samples were processed in the Aquatic Ecology Laboratory (LEA) of San Francisco de Quito University (USFQ). The ash-free dry mass (AFDM) was calculated with the weights of the dried and oxidized filters [69].

### Aquatic insect collection for the microcosm

**Fig 3:**
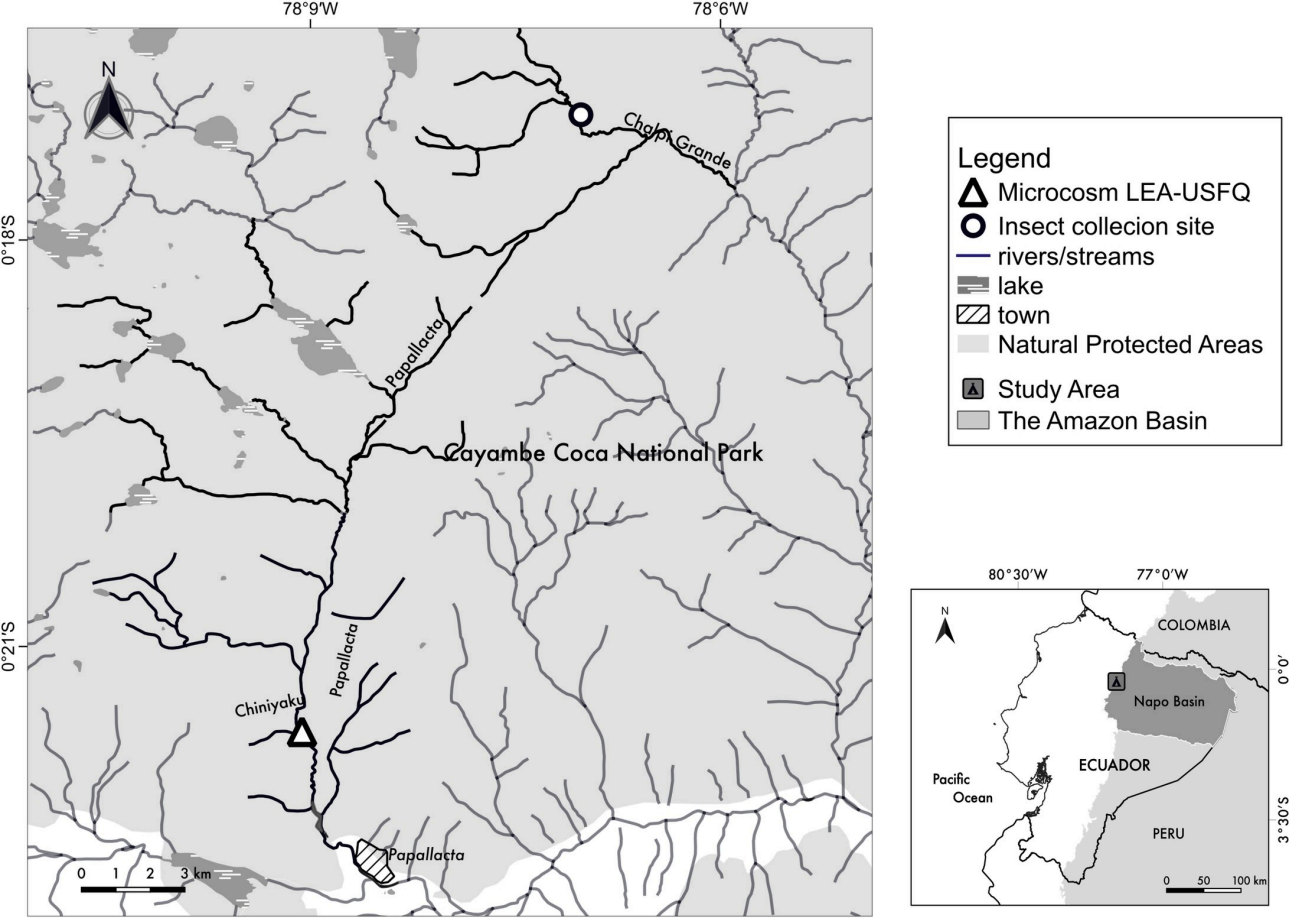
Experimental facility located in the Papallacta region, Napo Province, Ecuador. Insect collection site in the Cayambe Coca National Park and location of the microcosm facility. The rivers and streams in this area are part of the Napo subbasin, Amazon Basin.

Fieldwork was carried out during the early morning of February 1, 2019, at the PAP-129 stream in a tributary of the Chalpi Grande Basin (WGS84; 0.286921S -78.115419 W, 3684 m.s.a.l.). The site was located in the Cayambe Coca National Park in Napo Province of Ecuador (Fig 3), 16 km from the experimental facility. This stream is a high-elevation drainage river composed of water derived from precipitation and runoff in the páramo areas of the eastern flank of the Ecuadorian Andes [37]. Records of daily air temperatures in this area fluctuate from 5°C to 17°C, and precipitation is lowest from September to February [70]. The annual average temperature in streams from the Chalpi Grande watershed was 8.14°C (SD = 0.65) [71]. On the day of insect collection, the stream temperature was 9.9°C, oxygen saturation was 90.3%, and dissolved oxygen was 6.9 mg/L.

**Fig 4:**
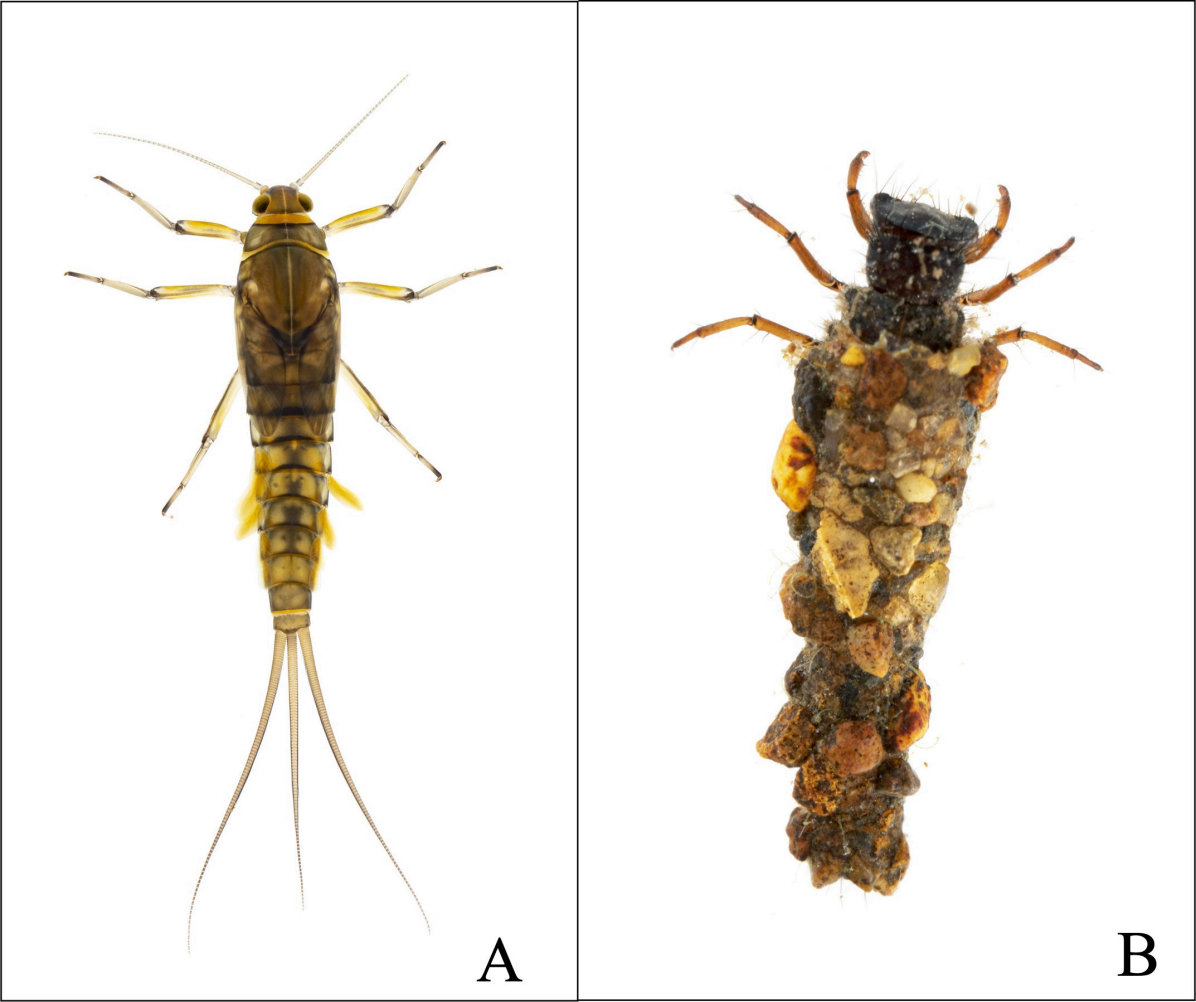
Larvae of aquatic insect species. (**A**) Mayfly larvae, *Andesiops peruvianus* (Ulmer, 1920). (**B**) Caddisfly larvae, *Anomalocosmoecus illiesi* (Marlier, 1962). Reprinted from [www.ex-situphotography.com/es/proyectos-publicaciónes/proyectos-publicaciones] under a CC BY license, with permission from [Vieira, J. ExSitu], original copyright [©ExSitu, all rights reserved].

We collected two aquatic insect species reported as the dominant species in high-elevation Andean streams in the Neotropics in general [46, 72–74], particularly in our fieldwork area [32, 36, 37, 75]. The first was a hemimetabolous species, *Andesiops peruvianus* (Ulmer, 1920) (Fig 4A), which has altitudinal ranges from 1000 to 4300 meters a.s.l in South America [45, 72, 76]. The second was the holometabolous species *Anomalocosmoecus illiesi* (Marlier, 1962) (Fig 4B), which in turn is restricted to high-páramo areas above 3000 m.a.s.l [36, 77]. These two species have been described to feed mostly on organic matter and algal resources [67, 68, 78, 79].

The collection was performed manually using a 250 μm mesh pore Suber-net to reduce potential insect stress and damage. Individuals were placed in plastic trays where they were isolated from the other aquatic taxa trapped in the net. Individuals were immediately placed in hermetic bags with natural stream water and transported in coolers with icepacks. Water pumps were placed inside the iceboxes until their arrival at the laboratory. Experimental species were stocked immediately upon arrival, as previous experiments reported a high mortality rate of mayflies when left acclimating for several days [53].

### Aquatic larval growth rate during the experiment

Previous studies used linear regressions to analyze the growth of traits such as body length and head capsule width (among others) as an estimate of biomass in aquatic invertebrates [80–82]. Such a length–mass relationship has been a powerful tool for addressing ecological questions related to metabolism and ecophysiology [81, 83]. We estimated larval growth by measuring body length in *Andesiops peruvianus* and head capsule width in *Anomalocosmoecus illiesi*. In addition, we measured forewing-pad length in mayfly larvae to characterize differences in wing growth [84]. Mayfly larval body length was measured from the anterior edge of the clypeus to the posterior edge of the last segment of the abdomen, and wing pads were measured from the posterior margin of the mesonotum to the posterior edge of the wing pad [56]. Head capsule width in caddisfly larvae was measured between the external borders of the forehead since this measurement has been suggested to be the most reliable metric for detecting changes in the development of trichopteran larvae and to determine instars. It is also highly related to body length in this species, making this measure more suitable for living larvae [85–88].

ImageJ software [89] was used to measure mayfly larvae using digital photographs taken by a Canon EOS Rebel-XS camera combined with an 18–55 mm EF-S lens. A total of 180 mayfly larvae were photographed in a dorsal position immediately after arrival at the microcosm facility. Each specimen was placed in a water drop inside a Petri dish. Photographs were taken in manual mode from 0.25 m with a tripod, where a white halogen lamp provided illumination. After measurements were taken, three nymphs of *Andesiops peruvianus* were placed in each fiberglass chamber (*n* = 180). Next, head capsule widths of *Anomalocosmoecus illiesi* were measured with an Olympus SZ51 stereomicroscope (lenses: WHSZ10X-H-22). Two were then placed into each chamber with mayfly larvae after measuring *Anomalocosmoecus illiesi* larvae (n = 120). We did not take photographs of this species to avoid excessive stress and possible death of the specimens outside their case building. All body sizes of surviving larvae were measured at the end of the experiment using identical species-specific methods.

### Growth rate estimation

The length–mass relationship was analyzed according to Benke et al. [81]. In brief, the dry mass of the aquatic insects was calculated from a linear equation:

$$\text{M}=a{\text{L}}^{b}:$$

where *M* is individual mass (mg). *L* is the linear measurement (mm) of a specific part of the body of the insect (e.g., head capsule width, body length).

*a* and *b* are logarithmic constants, coming from the slope of the linear equation generated from the previous two [83].

The final equation used for *Andesiops peruvianus* was extracted from the Baetidae family, [81] M = 0.0051* L ^***2*.*895***^, and for *Anomalocosmoecus illiesi*, the equation was M = 0.0072* L ^***2*.*56165***^, obtained from previous studies [88].

Then, growth rates (mg mg^-1^ day^-1^) were calculated based on the equation:

$$g=\frac{ln\left(\frac{DMf}{DMi}\right)}{t}$$

where *DMf* is the average mass (mg) of the individual at the end of the experiment, estimated from the previous linear equation. *DMi* is the average mass (mg) at the beginning of the experiment. *t* is the time-lapse of the experiment [90].

### Ethics statement

The Environmental Ministry of Ecuador granted Scientific Research Authorization No. 019-2018-IC-FAU-DNB/MAE to the Universidad San Francisco de Quito. In addition, Mobilization Permit No. 10-02-2019-VS-DPAN-MAE was issued. Authorization specifically allowed us to collect and transport larval individuals from Stream PAP-129 inside the Cayambe Coca National Park located in Napo Province in Ecuador. Preservation of the specimens at the end of the experiment was performed according to LEA-USFQ protocols (90% ethanol). The material was deposited in the LEA-USFQ aquatic insect collection.

### Statistical analysis

To determine if environmental variables had significant differences between temperature scenarios (TEMP: [S1], [S2], [S3]), we performed a one-way ANOVA. A Tukey post hoc test observed differences between scenarios and eta-squared (η^2^) was performed to observe how our temperature affected water chemistry. To detect differences in FPOM over time between temperature treatments, we used a mixed (between–within) two-way ANOVA. Two categorical variables were used: temperature scenarios (TEMP: [S1], [S2], and [S3]) (between groups) and the day of data collection (DAY: 1^st^, 5^th^, and 10^th^). A Bonferroni post hoc test was performed to detect the differences in both categorical variables.

Mean daily temperature and mean maximum and mean minimum temperatures were also calculated from data loggers set in the chambers of the three temperature systems (S1, S2, and S3) during the experiment [91]. A t test between the two data loggers situated in two chambers from the same temperature scenario was computed to verify the temperature’s precision within the treatment.

Larval mortality rates for both species were compared under each temperature scenario. A generalized linear model (GLM) was chosen to compare mortality rates. Initially, we calculated the number of deaths and survivors as a percentage within the *n* population under each temperature scenario. A response variable was generated as a two-vector factor with mortality and survivorship percentages. Then, a binomial GLM was used for proportion data [92]. Temperature scenarios were used in the model as the explanatory categorical variable (TEMP: [S1], [S2], and [S3]), and a one-way factorial analysis of deviance was performed. A quasi-binomial model was used to refit and adjust the model because overdispersion was found in the model [92]. In addition, species mortality influenced by any physicochemical variables in chambers during the experiment was examined. We performed a generalized linear model with binomial errors and logit function to observe how environmental variables influenced survival estimates. In the model, we defined survival as the proportion of individuals alive at the end of the experiment. Explanatory variables used in the model were those with statistically significant variables previously analyzed with a one-way ANOVA test.

To compare aquatic insect growth, we used measures of body size and mayfly and caddisfly larval dry mass, which we analyzed using a repeated measure two-way ANOVA [93]. We used body length and fore wing-pad length of *Andesiops peruvianus* and head capsule width of *Anomalocosmoecus illiesi* as response variables. Only the head capsule width of *Anomalocosmoecus illiesi* was log-transformed to achieve the normality requirements of the analyses. Temperature scenarios (TEMP: [S1], [S2], and [S3]) were used to compare the difference in body sizes between groups within days of the experiment (DAY: [1st] and [10th]). A Bonferroni post hoc analysis with a pairwise t test was used to observe differences between groups within days [93]. We did not perform ANOVAs on body size for mayflies that emerged (imagos) during the experiment due to the small number of individuals counted (*n =* 10). However, the mean body lengths and standard deviations were calculated.

We compared the growth rates estimated for each aquatic insect across temperature scenarios by applying one-way ANOVA in two separate models. For this analysis, one categorical variable (TEMP: [S1], [S2], and [S3]) was set in the model. A post hoc Tukey analysis with a pairwise t test comparison between temperature treatments was performed to test for differences due to climate change scenarios.

To compare the effects of temperature and FPOM on growth rates between species, we applied a linear mix model (LMM). For the analysis, mean FPOM values were calculated with samples collected in each temperature scenario during the experiment (three-day samples). Mean FPOM content was used as a categorical variable in ranges for each temperature scenario (FPOM: [fpom1], [fpom2], and [fpom3]. We included species (SP: [A.p] and [A.i]) in the model as a categorical variable to compare results between them. The microcosm chamber number was added as a random factor to observe the variance of each element in the model. Models were chosen according to the Akaike information criterion (AIC), and we plotted the residuals versus the fitted values from the model. All data were analyzed in the open-source software program R. Packages used for the analysis included rstatix, lme4, and ggplot [94–97].

## Results

### Environmental factors in the microcosm

Only dissolved oxygen varied significantly between temperature scenarios, while other environmental variables were not significantly different (Table 1). The average dissolved oxygen was highest at Temperature S1. The concentration declined significantly at S2 (*p* = 0.04) and S3 (*p* = 0.0005) with respect to S1 (Table 1). Phosphate, ammonium, nitrates, conductivity, total dissolved solids, and pH were similar among the temperature treatments (Table 1; see S1 Table for post hoc Tukey test).

**Table 1:** One-way ANOVA estimates (mean $\bar{\text{x}}$), standard deviation (SD), and *F value*s of physical and chemical parameters analyzed in water from chambers of the mesocosm at three temperature scenarios: S1 (control), S2 (+2.5°C), and S3 (+5°C). *p value* (95%). *η*^*2*^ generalized effect size (eta-squared).

	S1	S2	S3			
	$\bar{\text{x}}$ (S.D)	$\bar{\text{x}}$ (S.D)	$\bar{\text{x}}$ (S.D)	*F*_*2*,*15*_	*p*	*η* ^*2*^
NO_3_ (mg/L)	0.88 (0.34)	1.26 (0.57)	0.93 (0.3)	1.4	0.28	0.15
PO_4_ (mg/L)	0.35 (0.14)	0.24 (0.06)	0.23 (0.04)	2.9	0.08	0.27
NH_4_ (mg/L)	0.12 (0.10)	0.06 (0.04)	0.05 (0.03)	1.9	0.17	0.21
pH	6.8 (0.3)	6.9 (0.3)	6.8 (0.2)	0.5	0.62	0.06
Conductivity (μS)	79 (12.9)	86 (11.2)	81 (16.2)	0.4	0.65	0.06
TDS (ppm)	49.4 (8.1)	53.7 (7.3)	50.5 (10.2)	0.4	0.68	0.05
O_2_ (mg/L)	7.2 (0.3)	6.6 (0.4)*	6.1 (0.4) ***	12.1	***0*.*0007***	0.62
% O_2_	92.2	89.9	88.3	0.59	0.56	0.07

p<0.05*;

p<0.01**;

p<0.001***; TDS-total dissolved solids, O_2_-dissolved oxygen, % O_2-_Saturation

**Fig 5:**
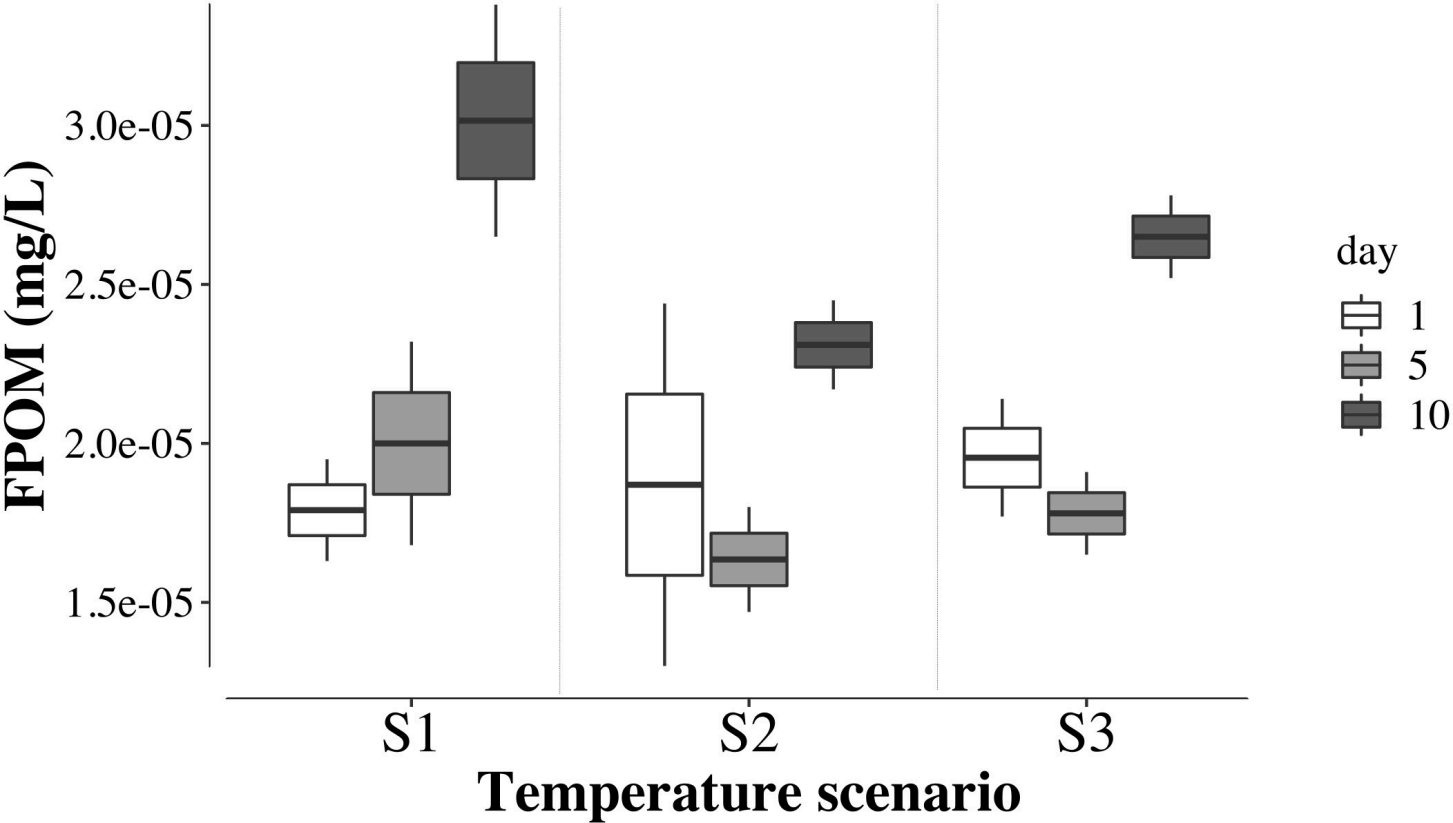
Fine particulate organic matter in three temperature scenarios. Boxplots of the fine particulate organic matter collected during the ten-day mesocosm experiment. The central line represents the median FPOM content. The lines above and below the box are the highest and lowest scores, respectively. The first and third quartiles are represented in the box. S1 (control), S2 (+2.5°C) and S3 (+5°C).

FPOM biomass showed that it varied independently from water temperature (TEMP: *F*_*2*,*3*_ = 1.2, *p =* 0.3) but varied between days of the experiment (DAY: *F*_*2*,*6*_ = 8.06, *p =* 0.02), with a pronounced increase on Day 10 (Fig 5). FPOM increased dramatically on the 10^th^ day of the experiment in all temperature treatments when it was almost double that of the 5^th^ day (*p =* 0.03) and 1^st^ day (*p =* 0.006) (Fig 5). There was no statistically significant interaction between the effects of temperature and the day of the experiment (TEMP x DAY: *F*_*4*,*6*_ = 0.5, *p =* 0.2), indicating that the temperature treatments were stable throughout the study (see S2 Table).

Pairwise t test comparisons of temperatures recorded from HOBO-data loggers in two chambers from S1-block showed no significant differences between them (*t*_*1*,*2318*_ = 1.91, *p =* 0.2); neither did the two chambers from S2-block (*t*_*1*,*2318*_ = 0.00008, *p =* 0.9), nor did chambers from S3-block (*t*_*1*,*2318*_ = 0.6, *p =* 0.4) throughout the 10-day study.

### Larval mortality rates

Temperature differences significantly affected mortality in *Andesiops peruvianus* (GLM: Df = 2, Res. Dev. = 1.5, *p<*0.05). The mean mortality rate of mayflies placed in the S1 chambers was 39%. There was an increase in mean mortality in S2 chambers (16%) and a significant increase in S3 chambers (22%) above control conditions (Table 2). In contrast, *Anomalocosmoecus illiesi* had lower mortality rates, and temperature did not significantly affect larval survival (GLM: Df = 2, Res. Dev. = 0.9, *p =* 0.2). The mean mortality rates in larvae from S1 were significantly lower (3%) than those from S2 (12%) and S3 (7%) (Table 2).

**Table 2:** Coefficients from the GLM (family = quasibinomial) of mortality rates for *Andesiops peruvianus* and *Anomalocosmoecus illiesi* in different temperature scenarios. Estimates were in link = *logit* with confident high (conf.high) and low (conf.low) values. Temp: S1 (control) S2 (+2.5°C) and S3 (+5°C). Significance coefficients (C I = 95%) are in bold.

term	estimate	S.E	Z-values	*p-*value	conf.low	conf.high
*Andesiops peruvianus*						
TempS1	-0.48	0.28	-1.70	0.09	-1.04	0.06
TempS2	0.68	0.39	1.73	0.09	-0.09	1.45
TempS3	0.95	0.40	2.40	***0*.*02***	0.18	1.74
*Anomalocosmoecus illiesi*						
TempS1	-3.66	1.00	-3.68	***<0*.*001***	-6.47	-2.16
TempS2	1.72	1.10	1.56	0.12	-0.14	4.62
TempS3	1.15	1.16	1.00	0.32	-0.92	4.11

The effect of the environmental variables on larval mortality was analyzed only with dissolved oxygen, which varied significantly between temperature scenarios (Table 1) and in combination with mean temperature (derived from data loggers). The model showed that the survival of *Andesiops peruvianus* larvae did not depend on dissolved oxygen alone (O_2_: LR-Chisq = 2.7, Df = 1, *p =* 0.09) and/or temperature in isolation (Temp: LR-Chisq = 2.7, Df = 2, *p =* 0.13), whereas the combined effects of dissolved oxygen and temperature had a significant impact on survival in mayfly larvae (O_2_ x Temp: LR-Chisq = 2.7, Df = 2, *p =* 0.01, AIC = 557.53) (Table 3). Mayfly survival was higher (62%, *p =* 0.11) in chambers from S1 with a mean of 9.2°C, where significantly higher dissolved oxygen was also found. In chambers with a mean 11.9°C (S2), there were lower dissolved oxygen levels, where survival was reduced to 45% (*p =* 0.07) of the total population. Significant mortality in mayflies (survival: 39%, *p =* 0.002) was found at 14.6°C, similar to lower dissolved oxygen quantities.

**Table 3:** Analysis of deviance of the GLM with a binomial distribution (link = *logit*.) of larval survival (%) influenced by dissolved oxygen (O2) and mean temperature (T) during the mesocosm experiment. Significant terms are in bold (CI = 95%).

	LR-Chisq	Df	*p* (>Chisq)	AIC
*Andesiops peruvianus*				
O_2_	2.78	1	0.09	557.5
Mean T	3.93	2	0.14	
O_2_ x T	9.09	2	***0*.*01***	
*Anomalocosmoecus illiesi*				
O_2_	0.04	1	0.82	550.7
Mean T	0.15	2	0.93	
O_2_ x T	0.05	2	0.98	

*Anomalocosmoecus illiesi* survival was not influenced by any variable in the model (dissolved oxygen and temperature), and the null model was chosen (AIC = 545.1; Table 3). In this experiment, survival in this species was higher than in mayflies, where more than 80% of the specimens survived all temperature treatments.

### Body size and dry mass

Repeated measures of two-way ANOVAs showed that the *Andesiops peruvianus* larval mean body length increased significantly throughout the experiment in all temperature scenarios (TEMP x DAY: *F*_2,71_ = 5.8, *p<* 0.0001). Temperature alone showed an effect on body length (TEMP: *F*_2,71_ = 9.6, *p<* 0.0001), and there was a difference in body length over time (DAY: *F*_1,71_ = 20.4, *p<* 0.001). Post hoc Bonferroni pairwise t tests indicated that body length increased from day one to ten in S1 (*p<*0.001) and S3 (*p<*0.0001) but not in S2 (*p =* 0.1). The pairwise t test between temperature scenarios showed no differences in individual lengths placed in S2 compared to the individuals in S1 on the first day (*p =* 0.82) (Table 4). However, individuals placed in S3 were smaller than those placed in S1 (*p<* 0.0001) and S2 (*p<* 0.0001) (Table 4). On the last day (10^th^) of the experiment, larvae showed no significant differences in body length between temperature scenarios (S1-S2: *p =* 0.3; S1-S3: *p =* 0.09; S2-S3: *p =* 0.6) (Table 4).

**Table 4:** Mean ($\bar{\text{x}}$), standard deviation (SD), and standard error (SE) of body length, wing-pad length and dry mass from *Andesiops peruvianus* and *Anomalocosmoecus illiesi* head capsule width and dry mass. S1 (control), S2 (+2.5°C) and S3 (+5°C). *n*, number of individuals measured.

Scenario	Day	$\bar{\text{x}}$	S.D	*n*	S.E
*Andesiops peruvianus*					
Body length *(mm)*					
S1	1	8.98	1.32	60	0.19
S1	10	9.93	1.05	35	0.18
S2	1	9.04	1.31	60	0.19
S2	10	9.62	1.11	17	0.27
S3	1	7.85	1.24	60	0.18
S3	10	9.44	1.02	22	0.22
Dry mass (*mg*)					
S1	1	0.83	0.12	60	0.018
S1	10	0.92	0.10	35	0.016
S2	1	0.83	0.12	60	0.018
S2	10	0.89	0.10	17	0.025
S3	1	0.72	0.11	60	0.017
S3	10	0.87	0.09	22	0.020
Wing-pads (*mm*)					
S1	1	2.15	0.75	60	0.11
S1	10	3.15	0.72	35	0.12
S2	1	2.31	0.69	60	0.10
S2	10	3.41	0.58	17	0.14
S3	1	1.84	0.77	60	0.11
S3	10	2.90	0.65	22	0.14
*Anomalocosmoecus illiesi*					
Head capsule width (*mm*)					
S1	1	1.24	0.36	40	0.06
S1	10	1.47	0.28	39	0.04
S2	1	1.24	0.33	40	0.05
S2	10	1.51	0.31	36	0.05
S3	1	1.26	0.35	40	0.06
S3	10	1.57	0.25	37	0.04
Dry mass (*mg*)					
S1	1	0.12	0.033	40	0.005
S1	10	0.14	0.026	39	0.004
S2	1	0.12	0.031	40	0.005
S2	10	0.14	0.029	36	0.005
S3	1	0.12	0.033	40	0.005
S3	10	0.15	0.023	37	0.004

In contrast, the interacting effects of temperature and time did not influence wing-pad length in *Andesiops peruvianus* and had a nonsignificant effect (TEMP x DAY: F_*2*,*71*_ = 2.59, *p =* 0.56). In contrast, temperature (TEMP: *F*_2,71_ = 5.3, *p<* 0.001) and time (DAY: *F*_1,71_ = 87.8, *p<* 0.0001) alone showed significant differences. A post hoc test with a pairwise t test analysis showed a significant increase in wing-pad length at the end of the experiment in all treatments (Table 4). Wing pads grew in size in all treatments throughout the experiment; S1-S3 (*p<* 0.0001). Because the initial body lengths for larvae in Treatment S3 were smaller, there were significant differences in wing-pad length among individuals in Treatment S3 when compared with Treatments S1 (*p<* 0.0001) and S2 (*p<* 0.0001) at the start of the experiment (Table 4). By the last day, differences were measured in the wing-pad length of individuals in S2 compared to S3 (*p =* 0.02) but not between S1 and S2 (*p =* 0.17) or S3 (*p =* 0.2) (Table 4).

Subimagos of *Andesiops peruvianus* emerged starting from day seven. Two individuals emerged from the S1 chambers and they had the largest body length ($\bar{\text{x}}$ = 10.2 mm, SD = 0.5), six individuals emerged from S2 chambers and they had a smaller mean body size compared to the control (S1) ($\bar{\text{x}}$ = 9.3 mm, SD = 1.1), and two subimagos from S3 had the smallest body length ($\bar{\text{x}}$ = 8.9 mm, SD = 1.1).

*Anomalocosmoecus illiesi* growth was independent of temperature (TEMP x DAY: *F*_2,109_ = 0.4, *p =* 0.6). Repeated ANOVAs of larval head capsule width showed no significant differences between temperature treatments (TEMP: *F*_2,109_ = 0.4, *p =* 0.6). Nevertheless, we observed an increase in larval size throughout the experiment such that the final sizes showed a 2-fold size increase in all treatments (DAY: *F*_1,109_ = 42.8, *p<* 0.0001) (Table 5). The post hoc analysis showed a significant increase in head capsule width in S1 (*p<* 0.005), S2 (*p<* 0.001) and S3 (*p<* 0.001) (Table 4). No differences in head capsule width sizes were detected on the first day between temperature scenarios (pairwise t test DAY-1: S1-S2: *p =* 1; S1-S3: *p =* 0.7; and S2-S3: *p =* 0.7) or at the end of the trials (pairwise t test DAY-10: S1-S2: *p =* 0.5; S1-S3: *p =* 0.1; and S2-S3: *p =* 0.4). No individuals emerged or pupated in this species.

**Table 5:** LMM performances of growth rates of *Andesiops peruvianus* and *Anomalocosmoecus illiesi* with mean FPOM as fixed factors and chamber as a random factor. Species [SP] was added as a covariate. The models tested were the null model, SP+FPOM without interaction, and SP x FPOM with single and interaction effects. The AIC best model is in bold.

Model	Df	AIC	BIC	logLik	deviance	Chisq	ChiDf	*p*(>Chisq)
Null	4	-610.49	-602.68	308.25	-616.49			
SP +FPOM	6	-614.39	-598.75	313.19	-626.39	9.89	3	0.02
SP x FPOM	8	**-615.47**	-594.63	315.74	-631.47	5.09	2	0.08

The dry mass of both species showed trends in RM-ANOVA similar to those of body size (see S3 Table for dry mass results).

### Aquatic larval growth rates

**Fig 6:**
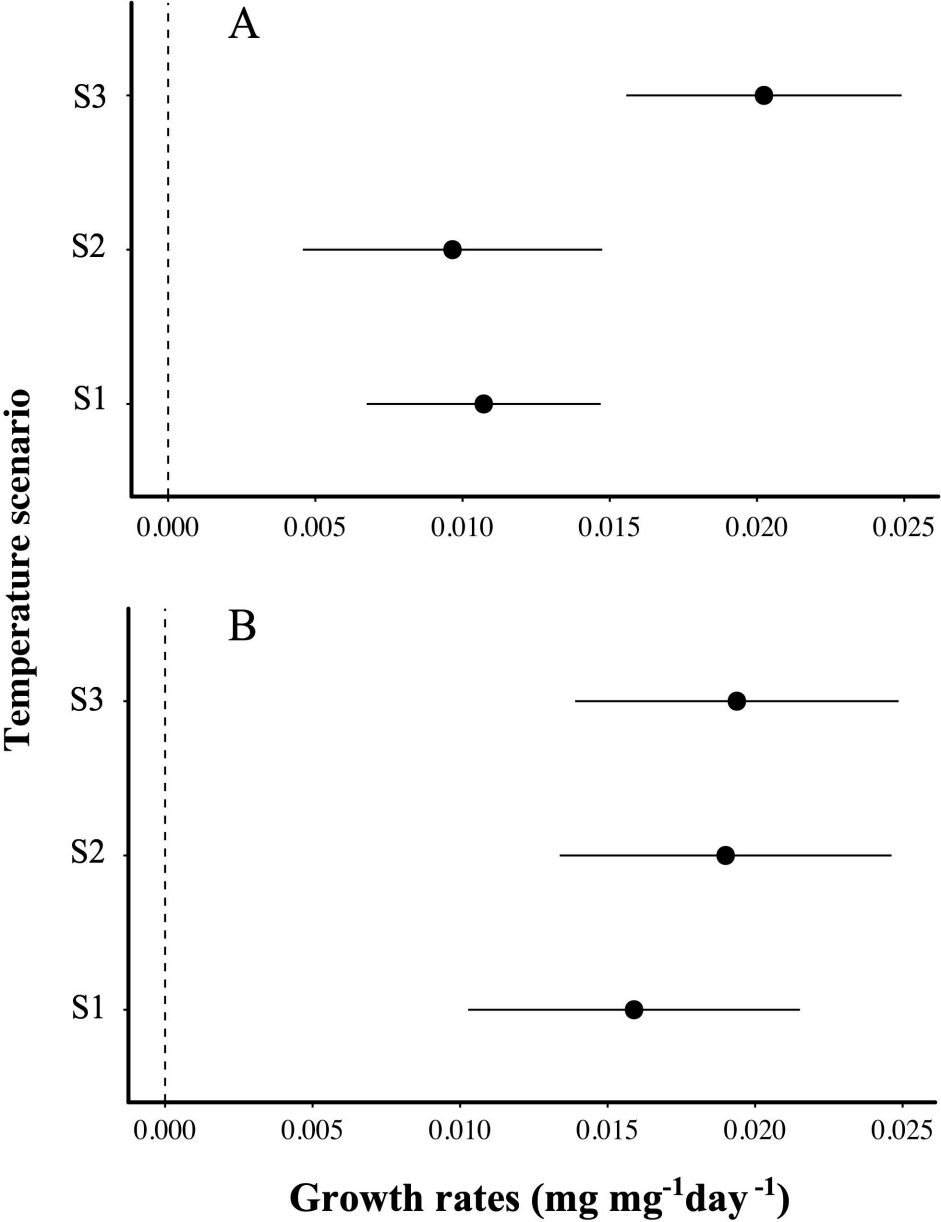
Aquatic insect growth rates subjected to rising temperatures. Growth rates of two aquatic insect species during a ten-day experiment. **(A).** *Andesiops peruvianus* growth rates (upper plot). **(B).** *Anomalocosmoecus illiesi* growth rates (bottom plot). Black dots are the mean growth rate. Lines represent confidence intervals (CI = 95%). The X-axis represents the estimated mean growth rate (mg mg mg^-1^ day^-1^). The Y-axis represents the temperature scenarios of climate change. Temp: S1 (control), S2 (+2.5°C), and S3 (+5°C).

Growth rates in *Andesiops peruvianus* were influenced by temperature (TEMP: *F*_3,39_ = 6.4, *p =* 0.004; Fig 6A). Post hoc analysis showed no differences in growth rates between individuals placed in S1 versus S2 (*p =* 0.7), but there was a significant difference between S1 and S3 (*p =* 0.003) and S2 and S3 (*p =* 0.004) (see Fig 6A). The mean growth rate of larvae placed in S1 was 0.011 mg mg^-1^ day^-1^. Larvae in S2 were similar to those in S1, growing on average 0.009 mg mg^-1^ day^-1^. However, larvae located in the S3 temperature chambers had significantly faster growth rates (*g* = 0.020 mg mg^-1^ day^-1^) (Fig 6A). Growth rates in *Anomalocosmoecus illiesi* presented no differences among temperature scenarios (TEMP: *F*_2,55_ = 0.46, *p =* 0.6; Fig 6B). A post hoc test revealed that larvae raised in S1 chambers had a 0.0159 mg mg^-1^ day^-^1 mean growth rate. Both S2 (g = 0.0190 mg mg^-1^ day^-1^. *p* = 0.71) and S3 (g = 0.0194 mg mg^-1^ day^-1^.) larvae had no significantly different mean growth rates compared to S1 (*p =* 0.64). Growth rates were similar between the S2 and S3 temperature treatments (*p =* 0.99) (see Fig 6B).

We performed three linear mix models (LMMs) between growth rates and the amounts of FPOM, with species [SP] as a covariate. The full model was chosen because AIC performed better than those with single effects (SP x FPOM; Table 5).

The LMM showed that growth rates differed significantly between species (SP: *F*_*1*,*48*_ = 5.16, *p =* 0.02). The mean growth rate of *Anomalocosmoecus illiesi* was faster (g = 0.018 mg mg^-1^ day^-1^) than that of *Andesiops peruvianus (*g = 0.013 mg mg^-1^ day^-1^). These differences in growth rates were not strongly influenced by FPOM (FPOM: *F*_*1*,*54*_ = 2.19, *p =* 0.7). Nevertheless, the full model showed differences in growth rates associated with mean FPOM amounts at each temperature scenario between species (SP x FPOM: *F*_*1*,*54*_ = 2.5, *p =* 0.08; Table 6). Mayflies placed in chambers S1 and S3 with greater amounts of mean FPOM grew faster (*p<*0.05; Table 6), while those placed in S2 with a smaller amount of FPOM had no significant growth rates (see Table 6). For the trichopteran larvae, growth rates at temperature S1 were significantly slower when compared to S2 and S3, where larvae of *Anomalocosmoecus illiesi* in S1 had slower growth rates associated with greater FPOM amounts (Table 6).

**Table 6:** The LMM was used to compare the growth rates of *Andesiops peruvianus* [A.p] and *Anomalocosmoecus illiesi* [A.i] with the mean FPOM during the microcosm experiment. Estimates were extracted from the best candidate model with fixed and random effects. Significant coefficients are in bold (CI = 95%). Temperature scenarios S1(control), S2 (+2.5°C), and S3 (+5°C). LRT: likelihood ratio test statistic; and logLik: logarithmic likelihood of random effect (chamber).

Fixed effects											
	Term		Estimate		*p-*value		Estimate				*p-*value
	FPOM (mg/L)		A.p (mg mg^-1^ day^-1^)				A.i (mg mg^-1^ day^-1^)				
S1	2.27E-05		0.0109		***0*.*02***		0.0158				***<0*.*001***
S2	1.94E-05		0.0091		0.062		0.0190				0.18
S3	2.13E-05		0.0205		***0*.*042***		0.0194				0.33
Random effects											
Term		Variance	SD	logLik		AIC		LRT	Df	Pr(>Chisq)	
Chamber		0.00034	0.006	279.7		-545.43		2.9	1	0.08	
Residual		0.00083	0.009								

Note: For the fixed effects, temperature was not included in the model since we used mean FPOM amounts extracted for each scenario [S1, S2, and S3]. It was integrated into this table to clarify the origin of the mean FPOM content. For the random factor, the variance of chambers was 29%. The number of parameters was 9.

## Discussion

### Larval mortality

In our microcosm trials, we assessed the effects of IPCC climate change scenarios on two aquatic insect species that are ecologically important and relatively abundant in high-Andean river ecosystems [34, 61]. Our results showed that *Andesiops peruvianus* larvae were most affected by temperature, and mortality increased in the warming scenarios. For this mayfly species, the higher mortality rate in both scenarios (S2 and S3) could indicate that increasing water temperatures was a significant source of stress, affecting population survival across a short-term 10-day trial (e.g., a heatwave), especially with an increase of +5°C, which increased mortality. Likewise, experiments with *Andesiops* found in the same study area showed higher mortality rates caused by increases in water temperature, pointing to a thermal metabolic sensitivity and/or physiological constraints in this genus [41, 54, 98]. Although we did not measure metabolic performance in our mayfly individuals, we observed low dissolved oxygen values in chambers at S2 and S3, which could indicate that mayflies struggled to survive more in a warmer environment with lower oxygen concentrations than those at ambient temperatures with more dissolved oxygen [7, 99]. Thus, oxygen could become a critical environmental trait. Previous studies stated that aquatic invertebrates (e.g., Odonata and Ephemeroptera species) had less capacity to exchange oxygen at larval stages at higher temperatures, showing population declines [54, 100, 101]. Our findings supported this, where we found mortality higher in climate change scenario S2 (+2.5°C) and even more in S3 (+5°C), where the temperature was coupled with low dissolved oxygen.

It has been hypothesized that aquatic insects from the tropics have developed lower thermal tolerances and therefore have less capacity to adapt to warmer water temperatures [102]. However, it has been observed that *Andesiops* from high-elevation streams (when compared with their low-elevation counterparts) have a strong acclimation ability when their thermal limits increase quickly (48-hour experiment). In our study, extending the period at which temperature increases were experienced (10-day experiment) produced higher mortality in *Andesiops*. Based on the previous study and our results, we argue that although *Andesiops* can withstand drastic temperature changes for a short time, when subjected to longer heatwaves as simulated by the S2 and S3 temperature scenarios in our experiment, we expect high levels of mortality.

In addition, high mortality rates have been observed in earlier larval stages at higher latitudes when subjected to increased temperatures [23, 25, 103, 104], similar to our observations of the individuals exposed to the S3 temperature scenario. Potentially, mortality in juveniles could be linked with rapid growth and aging, which would affect larval ability to repair any molecular damage associated with exposure to increasing temperatures, in which other studies have proposed alternative metabolic effects [105, 106]. Similarly, it has been observed that during molting, the mayfly larva *Cloeon dipterum* is critically sensitive to warmer temperatures, significantly increasing mortality [107].

In contrast, larvae of *Anomalocosmoecus illiesi* were more tolerant to warming temperatures and had an overall higher survival rate. The mortality rate of these holometabolous insects was lower than that of mayflies. Negative responses in this species may not be as drastic at the larval stage but could potentially be observed later in other physiological processes or when adults emerge. Previous studies performed with *Anomalocosmoecus* showed a high capacity to survive some levels of stressful adverse conditions in both laboratory trials and outdoor experiments [78, 98, 108]. Nevertheless, given the relatively short duration of our trial (10 days), we did not observe any effects (positive or negative) throughout the larval, pupal, or adult stages in our temperature experimental setup. Therefore, perhaps longer experimental studies are needed since its larval stage might last at least three months [88]. However, longer experiments are difficult to conduct, and a feasible option would be to conduct experiments focused on different life stages of this species.

### Body size, dry mass, and growth rates

In our experiment, *Andesiops peruvianus* increased length and body mass throughout the study period. However, we did not observe any differences in larval body length, mass, or wing-pad length in higher temperature scenarios in regard to the ambient conditions. Nonetheless, we could highlight our results regarding the mean body length, dry mass, and wing-pad length of mayfly larvae in +5°C chambers. Even though larvae were smaller at the beginning of the experiment, on the 10^th^ day, they reached almost identical body sizes (body and wing pad) as their counterparts reared at the lower temperatures in S2 and S1. The growth rates of larvae at +5°C were faster than those from the other temperature scenarios. These findings support the prediction that higher temperatures benefit this species’ early larval stages, as previous studies have suggested [10, 17, 58, 109]. Several experiments have found that mayflies have high growth rates at larval stages as temperature increases, resulting in smaller sizes at the mature stage (imago) [24, 110, 111]. Because we had relatively few individuals who emerged during our experiment, we could not infer whether temperature directly affected body size in adulthood. Although our number was low, we observed that adult mayflies in +5°C chambers had smaller body and wing sizes than those that emerged at temperatures in Experiments S1 and S2. The rapid growth of *Andesiops peruvianus* larvae placed in +5°C microcosms could additionally have further downstream consequences on morphological traits other than growth rates, which could affect adult mating success or fecundity [12]. Other studies have shown high mortality during juvenile development similar to our results in the +5°C treatment, increased risk of predation, and poor adult performance in other aquatic species, such as mayflies, caddisflies, stoneflies, and dragonflies [103, 104, 112, 113].

Although *Anomalocosmoecus illiesi* larvae did show a significant increase in their head width at the end of the experiment, contrary to our initial hypothesis, increased temperatures did not explain differences in growth, body size, or mass. We could not discover any relationships between larval growth and rearing temperatures based on the body size measurements at the larval stage. Nevertheless, it must be noted that our dry mass results were calculated based on Benkes’s [81] equation and not by actually weighing the specimens. Most likely, differences in body weight could have occurred. Mechanistic models have described how temperature regulates body size and growth in terrestrial holometabolous insects. They show how temperature affects physiological processes through metabolic reactions [114–116].

For example, it has been observed in insects, such as *Manduca sexta* (a moth) and *Drosophila melanogaster* (a fly), that the mechanisms by which temperature determines final body size are different [117, 118]. In *D*. *melanogaster*, when temperatures increased, larvae of the first instars stopped growing, remaining small, which was also perceived in adults. However, in *M*. *sexta*, increased temperatures did not affect first instars, and growth rates increased; however, there was a considerable reduction in body mass before the pupa stage, and adults were also smaller [119]. Our holometabolous insect, *Anomalocosmoecus illiesi*, may similarly respond to this model. If this were the case, lower body mass would be observed in the last instars of the larvae than at the pupal stage. However, further studies encompassing or focusing on their entire development are needed [88].

On the other hand, we observed a higher survival rate in this trichopteran species, indicating a more plastic response to increased temperatures during our experiment. Larvae increased their body size independently of the warmer water. The positive response to the increased temperature at the larval stage could be explained if this species’ metabolic traits related to growth were not affected by temperature (at the pupa or adult stage). Another possibility is that the development time of *Anomalocosmoecus illiesi* is longer than that of *Andesiops peruvianus*, and longer experimental trials or focused on different stages would be needed to elucidate the response to increasing temperatures of this species. For example, long life cycles have been found in other Limnephilidae species from Northern latitudes at similar temperatures, where larval and pupa instars lasted more than three months [85, 86, 120, 121].

Moreover, variations in the growth rates of aquatic insects can also be explained by characteristics other than temperature, such as the availability of food resources [25, 27]. Our results, for example, highlight that fine particulate organic matter was related to *Andesiops peruvianus* growth rates across all temperature scenarios but only for standard ambient temperatures for *Anomalocosmoecus illiesi*. These results could be related to the food-processing rates of different species in Andean streams, such as in our microcosm experiment. Larvae of *Andesiops peruvianus* could be more dependent on the energy derived from (FPOM) detrital energy sources to grow, a trend that has been described in studies of Andean rivers [67, 68]. However, the effect of FPOM on *Andesiops peruvianus* growth was not as pronounced, perhaps because this species complements its diet with other food resources (e.g., periphyton). This type of input energy source has been described in Ecuador’s high-elevation streams, especially those with no riparian vegetative cover and high penetration of light in the river channels [122], as was characteristic of our aquatic insect collection site. Although we provided tiles colonized with algae throughout the experiment, the quantity and/or quality of algae could not have been sufficient for *Andesiops peruvianus* larvae given the temperature increases in the S2 and S3 treatments. These changes in nutrition could have affected energy assimilation and life histories, as other studies have found with different groups of mayflies [25, 123].

Food intake of fine particulate organic matter did not affect the growth rates of *Anomalocosmoecus illiesi*. These results were an interesting observation because the energy requirements of this species do not rely only on fine particulate organic matter. Therefore, additional food sources, such as CPOM and algae, were provided on the colonized tiles, and perhaps some other aquatic insects that colonized the microcosms (from the Chiniyaku stream) filled their energy requirements to maintain their metabolism. In this way, *Anomalocosmoecus illiesi* increased its mean size more than *Andesiops peruvianus*, independent of temperature differences. Additionally, in outdoor experiments, it has been observed that this species is an excellent contributor to the processing of benthic CPOM in páramo streams [79]. Other caddisfly species have been found to change food preferences as a response to stressful conditions and/or as a plastic response to the availability of food resources in the environment [124, 125].

Further implications of both species growth rate and size could be related to increasing temperatures. If warmer water affects adult body size, traits such as reproduction and voltinism could also change. Nevertheless, as these species are multivoltine, it is difficult to know the impact of higher temperatures on individuals, as there are different overlapping cohorts.

## Conclusions

We conducted a novel experimental study on two aquatic insect species from high-elevation streams in the Andes. Our results suggest that global warming will affect Andean aquatic insect species in two important ways: growth rates and overall survival. We observed less thermal tolerance in hemimetabolous mayflies, *Andesiops peruvianus*, which could have potential implications for populations by perhaps limiting their ability to disperse, migrate or emerge due to significant mortality in the larval stages. *Anomalocosmoecus illiesi* showed higher plasticity to temperature changes. Other environmental factors could possibly interact with higher temperatures (e.g., droughts, increasing precipitation rates, and runoff), drastically changing environmental conditions and enhancing negative effects on metabolic processes, development, fecundity, and emergence. Neither of our aquatic insect species exhibited significant differences in body size or mass in warmer water when compared with the ambient water temperature. However, we could observe a significant difference in growth rates in the smallest average size of *Andesiops peruvianus*, which occupied the warmest (+5°C) microcosms, indicating that increased temperatures stimulate growth at this larval stage, although this likely results in smaller sizes in mature individuals. On the other hand, the effects of the growth rates on the metabolism of *Anomalocosmoecus illiesi* may only be apparent upon metamorphosis rather than throughout larval development. However, confirmation would require different experimental approaches.

Further research is needed in terms of aquatic insect metabolism for the different life histories of these two species to fully elucidate how temperature and future climate change conditions could affect them. Additionally, we suggest testing how additional stressors impact high-elevation Andean streams, such as nutrient enrichment, pesticides, and mining pollutants, among other environmental characteristics that could affect insect species in combination with climatic change conditions.

## Supporting information

S1 Table Tukey post-hoc test of environmental variables. (DOCX)

S2 Table Tukey post-hoc test of FPOM. (DOCX)

S3 Table Repeated measures two-way ANOVA of dry masses. (DOCX)
