# Supplementary material for: Effects of experimental warming on two tropical Andean aquatic insects
Source: PLoS One. 2022 Jul 27;17(7):e0271256. doi: 10.1371/journal.pone.0271256 (PMC9328556; doi:10.1371/journal.pone.0271256)
Supplement: S1 Table — (DOCX) [file pone.0271256.s001.docx]

**S1 Table. Results from tukey post-hoc test with pairwise comparison between temperatures scenarios from samples of environmental variables. S1(control) S2 (+2.5 ºC) and S3 (+5ºC).**

| term | group1 | group2 | estimate | conf.low | conf.high | *p*.adj | *p*.adj.signif |
| --- | --- | --- | --- | --- | --- | --- | --- |
| Nitrates |  |  |  |  |  |  |  |
| temp | S1 | S2 | 0.37 | -0.26 | 1.01 | 0.31 | ns |
| temp | S1 | S3 | 0.04 | -0.59 | 0.68 | 0.98 | ns |
| temp | S2 | S3 | -0.33 | -0.96 | 0.31 | 0.40 | ns |
| Phosphates |  |  |  |  |  |  |  |
| temp | S1 | S2 | -0.10 | -0.24 | 0.03 | 0.15 | ns |
| temp | S1 | S3 | -0.11 | -0.25 | 0.02 | 0.12 | ns |
| temp | S2 | S3 | -0.01 | -0.14 | 0.13 | 0.99 | ns |
| Amonium |  |  |  |  |  |  |  |
| temp | S1 | S2 | -0.06 | -0.15 | 0.04 | 0.31 | ns |
| temp | S1 | S3 | -0.07 | -0.16 | 0.03 | 0.19 | ns |
| temp | S2 | S3 | -0.01 | -0.11 | 0.08 | 0.94 | ns |
| Dissolved oxygen |  |  |  |  |  |  |  |
| temp | S1 | S2 | -0.64 | -1.25 | -0.03 | 0.04 | * |
| temp | S1 | S3 | -1.15 | -1.76 | -0.55 | 0.00 | *** |
| temp | S2 | S3 | -0.51 | -1.12 | 0.10 | 0.11 | ns |
| TDS |  |  |  |  |  |  |  |
| temp | S1 | S2 | 4.27 | -8.71 | 17.2 | 0.67 | ns |
| temp | S1 | S3 | 1.06 | -11.9 | 14.0 | 0.98 | ns |
| temp | S2 | S3 | -3.20 | -16.2 | 9.78 | 0.8 | ns |
| pH |  |  |  |  |  |  |  |
| temp | S1 | S2 | 0.13 | -0.2 | 0.49 | 0.60 | ns |
| temp | S1 | S3 | 0.05 | -0.3 | 0.41 | 0.92 | ns |
| temp | S2 | S3 | -0.08 | -0.45 | 0.27 | 0.83 | ns |
| Conductivity |  |  |  |  |  |  |  |
| temp | S1 | S2 | 7.09 | -13.30 | 27.50 | 0.65 | ns |
| temp | S1 | S3 | 1.74 | -18.60 | 22.10 | 0.97 | ns |
| temp | S2 | S3 | -5.35 | -25.70 | 15.00 | 0.78 | ns |
| %O2 |  |  |  |  |  |  |  |
| temp | S1 | S2 | 0.57 | 5 | 0.59 | 1.0 | ns |
| temp | S1 | S3 | 0.94 | 5 | 0.38 | 1.0 | ns |
| temp | S2 | S3 | 1.32 | 5 | 0.24 | 0.73 | ns |
|  |  |  |  |  |  |  |  |
