# Supplementary material for: Effects of experimental warming on two tropical Andean aquatic insects
Source: PLoS One. 2022 Jul 27;17(7):e0271256. doi: 10.1371/journal.pone.0271256 (PMC9328556; doi:10.1371/journal.pone.0271256)
Supplement: S2 Table — (DOCX) [file pone.0271256.s002.docx]

**S2. Table. Results from Tukey post-hoc test with pairwise comparison between amounts of FPOM. Temperature scenarios: S1(control) S2 (+2.5 ºC) and S3 (+5ºC) and day of the experiment.**

| term | group1 | group2 | estimate | conf.low | conf.high | p.adj | p.adj.signif |
| --- | --- | --- | --- | --- | --- | --- | --- |
| temp | S1 | S2 | -0.000003 | -0.000012 | 0.00000485 | 0.56 | ns |
| temp | S1 | S3 | -0.0000014 | -0.0000095 | 0.00000675 | 0.89 | ns |
| temp | S2 | S3 | 0.000002 | -0.0000063 | 0.0000101 | 0.81 | ns |
| day | 1 | 5 | -0.0000007 | -0.0000062 | 0.00000489 | 0.95 | ns |
| day | 1 | 10 | 0.000008 | 0.0000023 | 0.0000134 | 0.006 | ** |
| day | 5 | 10 | 0.000008 | 0.0000029 | 0.0000141 | 0.003 | ** |
