# Supplementary material for: Effects of experimental warming on two tropical Andean aquatic insects
Source: PLoS One. 2022 Jul 27;17(7):e0271256. doi: 10.1371/journal.pone.0271256 (PMC9328556; doi:10.1371/journal.pone.0271256)
Supplement: S3 Table — (DOCX) [file pone.0271256.s003.docx]

**S3. Table. Results from repeated measures two-way Anova of dry mass of *Andesiops peruvianus* and *Anomalocosmoecus illiesi***

| Effect | D.Fn | D.Fd | F | *p* | *p<.*05 | Effect size |  |
| --- | --- | --- | --- | --- | --- | --- | --- |
| *Andesiops peruvianus* dry mass | | |  |  |  |  | |
| temp | 2 | 71 | 9.63 | 0.00 | * | 0.13 |  |
| day | 1 | 71 | 20.43 | 0.00 | * | 0.11 |  |
| temp:day | 2 | 71 | 5.83 | 0.01 | * | 0.07 |  |
| *Anomalocosmoecus illiesi* dry mass | | |  |  |  |  | |
| temp | 2 | 109 | 0.63 | 0.53 |  | 0.006 |  |
| day | 1 | 109 | 42.78 | 0.00 | * | 0.167 |  |
| temp:day | 2 | 109 | 0.44 | 0.65 |  | 0.004 |  |
